# Balance Wood, a Bilateral Ankle-Controlled Serious Game for Healthy Adults: 2-Stage Cross-Sectional Development and Usability Study

**DOI:** 10.2196/96617

**Published:** 2026-09-25

**Authors:** Yuqi Zhang, Junichi Yamamoto, Tianyi Han, Qingwei Song, Keiko Kasamatsu, Naoyuki Kubota

**Affiliations:** 1 Department of Mechanical Systems Engineering, Graduate School of Systems Design, Tokyo Metropolitan University, 6-6 Asahigaoka, Hino-shi, Tokyo, 1910065, Japan, 81 42-585-8441

**Keywords:** healthy adults, ankle control, serious game, exergame, usability, user experience, gameplay-derived signals

## Abstract

**Background:**

Ankle mobility and control contribute to balance and everyday movement throughout adulthood, yet are usually assessed through repetitive, abstract tasks that reveal little about movement production. Serious games can make repeated tasks engaging, but most report only end point scores rather than continuous gameplay signals, and few establish system usability. Developing and evaluating such a system in healthy adults is a necessary first step.

**Objective:**

This study aimed to develop Balance Wood, a bilateral ankle-controlled serious game in which seated players tilt a device through ankle flexion-extension to control on-screen play, to describe the system and the signals it records, and to evaluate its usability, safety, and user experience in healthy adults.

**Methods:**

In this two-stage cross-sectional development and usability study, healthy adults without lower-limb injury or balance-affecting neurological or vestibular disorders completed single-session repeated-measures procedures. In stage 1, a total of 40 adults—20 older (mean age 73.0, SD 5.9 y; 10 women) and 20 younger (mean age 28.1, SD 3.2 y; 4 women) adults—completed 3 fixed-order difficulty levels in 1 session (exploratory signal analysis). In stage 2, a total of 18 younger adults (mean age 24.6, SD 3.9 y; 5 women) completed the levels in counterbalanced order. Usability was assessed with the System Usability Scale (0‐100); use impressions and consciously used body parts with Likert and open-ended items; and device tilt-angle accuracy against a digital goniometer.

**Results:**

The mean System Usability Scale score was 81.5 (SD 11.2; 95% CI 76.0‐87.1; Cronbach α=.74), above the benchmark of 68, indicating good perceived usability. Mean ratings were 2.11 (SD 1.32) for safety concern (95% CI 1.45‐2.77) and 1.67 (SD 1.03) for pain (95% CI 1.15‐2.18). Ankle use was highest at 4.94 (SD 0.24), exceeding knee 2.50 (SD 1.29), hip 2.00 (SD 1.24), and trunk 2.17 (SD 1.15) by Wilcoxon signed-rank tests (*P*<.001). Device readings agreed closely with the goniometer, with a mean absolute error of 0.66°, an intraclass correlation coefficient of 0.999, and 95% limits of agreement of −0.68 to 1.70°. In stage 1, all measures were computable for the 40 participants, with typical amplitudes of 20.5° to 27.2° and stable speed in both groups. In stage 2, repeated-measures analyses (α=.05) did not detect statistically significant differences across difficulty conditions or presentation positions (all *P*>.13).

**Conclusions:**

In healthy adults, Balance Wood was usable, well-accepted, low-risk, and provided accurate device-level tilt measurement with predominantly ankle-based control while continuously recording gameplay signals. It is a low-cost, bilateral, ankle-specific control interface with continuous signal capture into 1 easy-to-use device. Its contribution is a reproducible, usability-tested platform with characterized device accuracy, and formally defined candidate measures awaiting validation. Once validated, it could support home- or community-based recording; in the near term, it is a research tool.

## Introduction

Ankle and foot motor control contribute to postural adjustment, weight transfer, and everyday movement throughout adulthood [1,2]. This control is graded and dynamic: ankle dorsiflexion force control shows reduced accuracy and greater variability in older than in younger adults [3,4], paralleling age-related declines in ankle proprioceptive acuity [5], and it depends on feedback about the ongoing movement [6]. Standing, weight transfer, and gait rely on both ankles acting together, yet most paradigms probe ankle control unilaterally, and age-related changes in bilateral force control—evident across effectors [7]—remain comparatively underexplored at the ankle. Characterizing graded, bilateral ankle control therefore calls for tasks that go beyond range of motion or strength alone [8]. Conventional clinical assessments, however, rely on abstract, repetitive tasks and report only maximum thresholds or static holding times, lacking the engaging context needed to capture continuous motor responses; when movement goals are unclear, confidence and the willingness to remain physically active may also decrease [9]. A practical need therefore exists for engaging systems that record interpretable signals of ankle-control behavior.

Serious games and exergames offer a promising alternative and appear feasible and acceptable across adult populations, from younger adults [10] to older adults [11-21], though a gap remains. Most existing systems are evaluated through overall training effectiveness, fall-related outcomes, or usability questionnaires, and report only end point scores such as total points or task success rather than the continuous movement signals generated during play. Recent work highlights the value of in-game sensing to capture movement during the task itself [22-24], and some serious games now derive interpretable motor metrics directly from gameplay signals—for example, quantifying movement slowness in Parkinson disease or upper-limb motor performance [25,26]. Such measures nonetheless remain less developed than broad outcome reporting [13,16,19].

This direction also aligns with Moonshot Goal 3, a Japanese government-led research and development initiative on long-term human support through the coevolution of AI and robots in real environments [27,28], within which support systems should accumulate structured behavioral data and capture fine-grained changes in movement patterns over time. We therefore developed Balance Wood, a serious game for the continuous signal recording of bilateral ankle control during gameplay. Participants control the tilt of a virtual wooden tub through slow forward and backward ankle movements, with intuitive physics-based feedback that conveys direction, movement size, and movement pace through changes in the water state.

To characterize the use and signal acquisition properties of Balance Wood, this study was structured as a 2-stage cross-sectional development and usability evaluation in which each participant was assessed in a single session. The aims were twofold: (1) to describe initial system use and signal-derived descriptive characteristics in a broader-age adult sample (stage 1, n=40 healthy adults across two age ranges); and (2) to systematically evaluate the system’s behavior under counterbalanced task order and its self-reported usability and user experience in a focused younger-adult sample (stage 2, n=18). A bench-level sensor calibration test of the embedded inertial measurement unit was additionally conducted. This study does not aim to establish measurement validity, demonstrate clinical utility, or test inferential hypotheses about age-related differences. The findings are intended to support future hypothesis-driven and clinical investigations of ankle-controlled serious games.

## Methods

### Study Design Overview

This 2-stage cross-sectional development and usability study used single-session repeated-measures procedures in both stages. Stage 1 was a fixed-order, within-subjects exploratory data acquisition study with 40 participants (20 older and 20 younger adults), characterizing the time-series signal output of Balance Wood and the descriptive utility of the proposed signal-based measures across trials of varying difficulty. Stage 2 was a counterbalanced supplementary study in an independent sample of 18 younger adults. Reporting of the usability evaluation follows the American Psychological Association Journal Article Reporting Standards for quantitative research, as reflected in the Methods subsection headings below.

### Ethical Considerations

**Figure 1.**
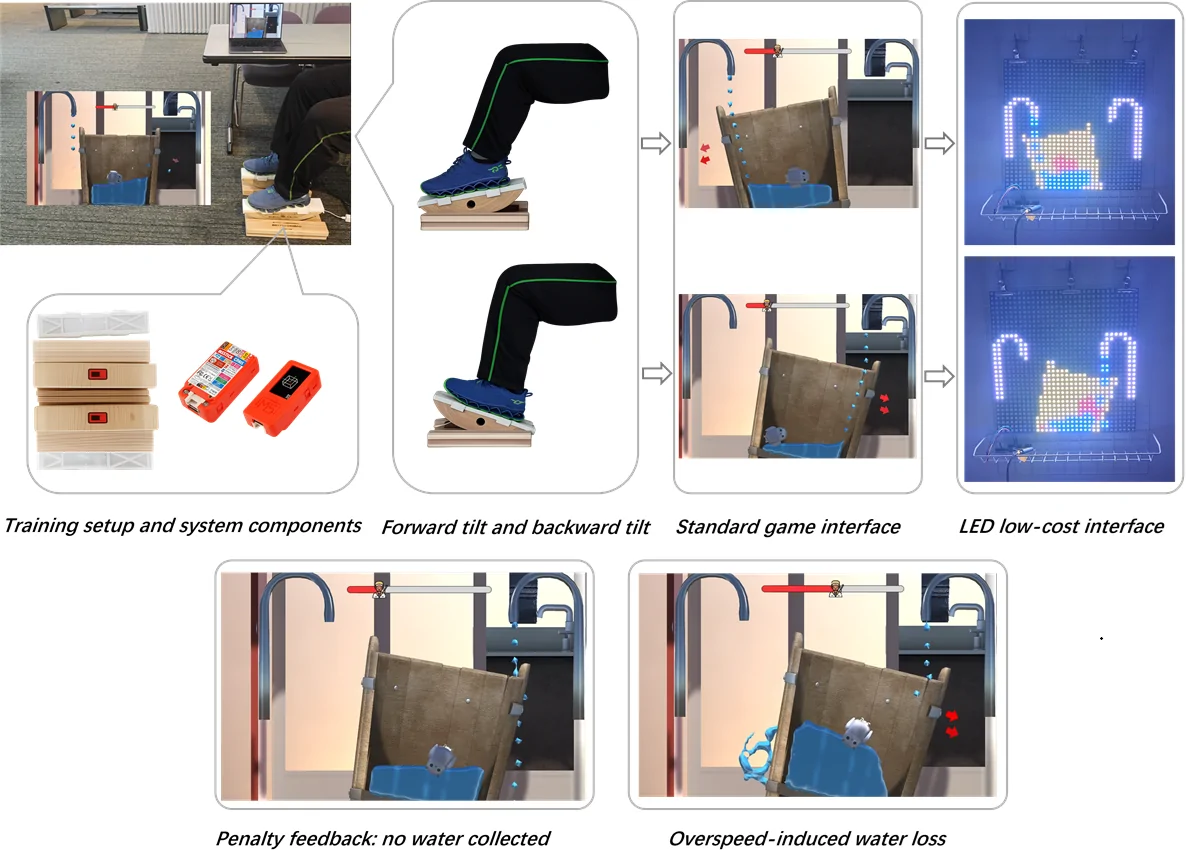
System overview and interface of Balance Wood. Upper left: real-use scenario, with 1 foot on each unit; each unit contains an embedded M5StickC PLUS (M5Stack Technology Co, Ltd) module that senses tilt angle and transmits signals over Wi-Fi. Middle left: forward tilt (top) and backward tilt (bottom) of a unit. Middle right: standard screen-based game interface. Upper right: low-cost LED matrix using the same directional control logic. Lower left: no-water-collected state (insufficient tilt or penalty-related return to neutral). Lower right: water leakage from movement exceeding the angular-velocity threshold. Negative values denote forward tilt and positive values denote backward tilt.

Both stages of this study were approved by the Research Ethics Committee of the Hino Campus, Tokyo Metropolitan University (R8-019). All participants in stages 1 and 2 provided written informed consent before participation; the stage 2 procedure (counterbalanced trial-order presentation and questionnaire administration) was covered under the same approved protocol. Before analysis, all recorded data were anonymized by the research team by replacing participant identifiers with numerical codes, and neither the analysis dataset nor this report contains any personally identifiable information. This study was conducted in accordance with the Declaration of Helsinki. Older-adult participants in stage 1 each received JP ¥6000 (approximately US $39, based on an exchange rate of approximately JP ¥153 per US dollar during the October-November 2025 data-collection period) as compensation; younger-adult participants in stage 1 and all stage 2 participants received no financial compensation. All images and videos in this paper and supplementary materials were reviewed to ensure that no study participant can be identified. The individual shown in Figure 1 and in Multimedia Appendices 1 and 2 is an author of this study, who provided written informed consent for the publication of their identifiable image and videos; no other identifiable individual appears in this paper or supplementary materials.

### Balance Wood System

Balance Wood is a bilateral system for the tilt-angle signal analysis and continuous signal recording of ankle control during gameplay, converting coordinated foot-tilt input into control signals for an interactive serious game. The system consists of 2 Balance Wood units placed under the feet of the participant, a host computer running the game, and a visual feedback implementation (Figure 1). In the standard configuration, the feedback is presented via a screen-based game. This design follows previous balance-oriented exergame work [11,12,16].

A low-cost alternative based on an LED matrix, operating without a dedicated host computer or display screen, was also developed [29-33]; it was not used in the usability evaluation reported here. Video demonstrations of the screen-based and low-cost LED versions in use are provided in Multimedia Appendices 1 and 2, respectively.

Each Balance Wood unit comprises a tilting wooden device with an embedded M5StickC PLUS (M5Stack Technology Co, Ltd) module mounted at the center. This embedded module simultaneously serves 2 functions: angle sensing and wireless communication. Specifically, each unit measures the respective tilt state and transmits the signal via Wi-Fi. The host system receives the signals from the 2 Balance Wood units through User Datagram Protocol communication. The transmitted value is the roll component of the attitude estimate produced by the module’s on-board sensor-fusion routine, which corresponds to the sagittal-plane tilt of the platform. Denoting the left- and right-unit tilt angles at time t as $\theta_{L}(t)$ and $\theta_{R}(t)$, the host computes the combined control angle as their signed sum:


$$\theta_{c}(t)=\theta_{L}(t)+\theta_{R}(t).$$

No other component angle enters the calculation, and no filtering, weighting, or dead band is applied. $\theta_{c}(t)$ is the variable that drives the game and, after the display scaling is reversed, is the variable used for all analyses reported here.

The attitude estimate is derived from the accelerometer and gyroscope signals of the module’s embedded inertial measurement unit. As low-cost micro-electro-mechanical-systems inertial sensors carry a fixed mounting and 0-point offset, an initial alignment (zeroing) was performed with the device flat before each session, so that all recorded signals were referenced to 0° [34]. Signals were sampled at approximately 9.5 Hz. Full hardware and signal-acquisition specifications are provided in Multimedia Appendix 3.

As the control variable is the signed sum of the 2 units, same-direction tilts from the 2 feet add, whereas opposite-direction tilts partially cancel; the interface therefore encourages coordinated bilateral movement. This simplified bilateral design lowers the difficulty of understanding how to operate the game [35-37].

In the recorded tilt-angle signal, forward tilt is represented by negative values and backward tilt by positive values; all main outcomes are described in terms of the forward and backward directions. All recorded signals correspond to the physical inclination of the foot device. For display purposes only, the host system applies a fixed 3/5 scaling factor to the on-screen bucket, so displayed and physical angles are not interchangeable: a displayed angle of 11° corresponds to approximately 18.33° of physical device tilt (Multimedia Appendix 3). The recorded data file stores the display-scaled values; the factor was divided out before analysis, so unless otherwise stated, all angular values reported here are unscaled physical angles.

The recorded signal represents the ankle-controlled tilt angle of the Balance Wood device, generated as participants drove the device through ankle flexion-extension. This device-level angle reflects ankle-driven control behavior rather than a direct anatomical measurement of ankle joint range of motion; the relationship between the device tilt and the underlying ankle joint angle is mediated by the foot-device interface and was not separately calibrated in this study.

### Task Interface and Display Implementations

In the standard screen-based implementation, a bucket is displayed in the center of the game interface, with a water source on each side. Water flows randomly from either the left or the right source, and each flow lasts for a randomly selected integer duration between 7 and 12 seconds. Participants are required to tilt the bucket toward the active source to collect the incoming water. Forward tilt corresponds to tilting the bucket to collect water from the left source, whereas backward tilt corresponds to tilting the bucket to collect water from the right source. The interface was designed so that the movement goal could be understood from the visual task outcome rather than from verbal instruction alone. This follows previous exergame work emphasizing intuitive, movement-oriented feedback [13,15,18,38-42].

In both display implementations, successful directional control allows the bucket to collect water from the currently active source; the low-cost version retains the identical bilateral ankle-input structure and directional control principle, consistent with compact wearable and embedded sensing approaches in rehabilitation and functional evaluation [14,31-33,43]. If the bucket is not tilted sufficiently toward the active source, water cannot be collected, and movement that is excessively fast causes water to leak. These 2 visual outcomes let participants judge their own directional accuracy and speed regulation from the state of the game.

### Adaptive Challenge-Penalty Mechanism

To elicit sustained, near-maximal excursions rather than passive low-effort movement, the system applies a direction-specific adaptive challenge-penalty rule, tracked independently for the forward and backward directions. This design logic follows broader principles of interactive systems, where a task must balance guidance and challenge to elicit a range of movements from the user [17,19,44-46]. The rule arms for a given direction only once the displayed bucket tilt reaches 11°, a playability threshold determined empirically during development as the lower bound at which the water-flow simulation both engages water collection and retains the collected water; it disarms when the movement returns close to the neutral position. Once armed, maintaining the movement at or above a fixed proportion of the current maximum peak avoids a penalty, whereas failing to do so returns the displayed bucket smoothly toward the midline; sustained performance at that criterion raises the peak threshold for the direction, subject to an observation window that reverts the increase if the criterion is not maintained. The penalty is applied to the displayed bucket angle only: the recorded signal always corresponds to the actual device tilt of the participant and never includes the penalty offset, and no score penalty or change to the water-flow rate is applied. The complete rule set is given in Multimedia Appendix 3, and the numeric value of every parameter is listed in Table S1 in Multimedia Appendix 3.

### Study Design

The performance of each participant was examined across 3 consecutive Balance Wood trials, and the resulting time-series signals were described using the proposed exploratory measures. The dataset comprised 40 participants: an older-adult group (n=20) and a younger-adult group (n=20). Including both age groups allowed the system to be exercised across a range of baseline movement capabilities.

The 3 formal trials differed inherently in difficulty, linked directly to the size of the falling water drops: lower difficulty meant larger drops and easier collection. Trial 1 was the easy condition; trial 2 was the medium condition, preset so that completing all water collection required close to 3 minutes, although faster participants could finish 10 to 20 seconds earlier; trial 3 was the high-difficulty condition, intentionally designed so that the task could not be fully completed even with maximal effort, to obtain a complete dataset of ankle behavior under maximal challenge. All 3 conditions ran under the same maximum duration of 180 seconds, and each trial ended at whichever came first: completion of the water-collection target for that condition or expiry of the 180-second limit. As completion requirements differed across trials, raw duration and holding time in seconds were not treated as direct evidence of performance; the analysis instead used the extracted signal-based measures.

### Inclusion and Exclusion Criteria

Participants were healthy adults who were able to perform repeated ankle flexion-extension safely and to provide written informed consent. Individuals with any acute or chronic condition that could interfere with safe participation—including lower-limb injury or pain, neurological or vestibular disorders affecting balance, or any contraindication to light physical activity—were excluded. For the older-adult group, participants were community-dwelling older adults; individuals unable to understand the task instructions or to provide informed consent were also excluded.

### Sampling Procedures

The younger-adult groups (stages 1 and 2) were recruited by convenience sampling from the Tokyo Metropolitan University community (Hino Campus) and the associated laboratory community, whereas the stage 1 older-adult group was recruited from the general community with support from the JST (Japan Science and Technology Agency) Moonshot Research and Development program (Moonshot Goal 3; JPMJMS2034). Data were collected at the Hino Campus of Tokyo Metropolitan University. In stage 1, the older-adult group was assessed between October and November 2025, and the younger-adult group between February and March 2026; stage 2 was conducted in June 2026.

### Sample Size, Power, and Precision

As a development and usability study, the sample sizes were chosen to be appropriate for early-stage usability evaluation rather than for confirmatory hypothesis testing. No formal a priori power analysis was conducted because the study is descriptive and exploratory in nature and is not designed to test inferential hypotheses; accordingly, the reported inferential statistics are interpreted as exploratory and are reported with CIs to convey precision. Stage 1 included 40 participants (20 participants per age group), and stage 2 included 18 younger adults, consistent with sample sizes commonly used in the usability and feasibility studies of serious games.

### Participant Characteristics

Only age and sex were collected; no other anthropometric or experience variables were recorded. Characteristics for all 3 groups are summarized in Table 1.

**Table 1.** Participant characteristics by study stage and age group (stage 1: single-session exploratory acquisition; stage 2: counterbalanced supplementary study).

	Stage 1, older adults (n=20)	Stage 1, younger adults (n=20)	Stage 2, younger adults (n=18)
Age (years)			
Mean (SD)	73.0 (5.9)	28.1 (3.2)	24.6 (3.9)
Range	60‐82	23‐35	21‐35
Sex, n (%)			
Female	10 (50)	4 (20)	5 (28)
Male	10 (50)	16 (80)	13 (72)

### Standardized Setup and Positioning

All sessions used an identical seated setup, standardized by target posture rather than by fixed equipment dimensions. Participants sat upright with the thighs approximately horizontal and both legs directed forward, so that hip flexion was approximately 90°, with the knees flexed to approximately 90°, the shanks hanging vertically, and each foot placed at the center of its Balance Wood unit; in this position, the platform was horizontal and was defined as 0° at session zeroing. Chair height was adjustable from 45 to 60 cm and was set to obtain this posture. Each unit was mounted on a movable wooden base board that permitted rotation in the sagittal plane only, mechanically preventing mediolateral tilt; with the boards in contact, the center-to-center distance between units was 13 cm, widened to approximately 22 cm, or at most 30 cm, for participants requiring a wider stance. All distances were measured directly. Participants wore their own shoes, which were required to be flat-soled, and the feet were not strapped or otherwise fixed to the platforms. Movement outside the ankle was not mechanically constrained; participants were instructed to maintain this seated posture and to control the device with the ankles.

### Experimental Procedure

Before the 3 formal trials, participants completed a low-difficulty practice session in which the instructor explained the game content, operating procedure, and precautions while observing performance in real time; formal testing began once the participant was confirmed to have understood the operation. Participants then completed the 3 trials consecutively in a fixed order (trial 1 to trial 2 to trial 3), and the system automatically recorded the tilt-angle signals of the device throughout testing [42,47].

### Stage 2 Procedure: Counterbalanced Supplementary Study

#### Overview

Stage 2 extended the stage 1 protocol in 2 respects. First, the 3 trials were administered in counterbalanced order, separating task difficulty from order effects such as motor learning, adaptation, and fatigue. Second, a standardized usability instrument and a structured feasibility questionnaire were added.

#### Counterbalanced Balance Wood Trials

Each participant completed the same 3 Balance Wood trials used in stage 1 (trial 1: easy; trial 2: medium; trial 3: high difficulty), but in counterbalanced order following a Latin square design. With 3 trial conditions, the full counterbalancing structure consists of 6 possible orderings (1-2-3, 1-3-2, 2-1-3, 2-3-1, 3-1-2, and 3-2-1). The 18 participants were assigned in equal numbers (3 per ordering) such that all 6 orderings were completed 3 times across the sample. Assignment to ordering followed a prerandomized sequence determined before recruitment.

The task interface, the adaptive challenge-penalty mechanism, and all system parameters were identical to stage 1. As in stage 1, brief practice was provided before the formal trials, and rest periods of at least 1 minute were provided between trials. The near-limit holding threshold used in the holding-proportion measures was defined per participant exactly as in stage 1, preserving within-participant comparability across the 2 stages.

#### Posttask Questionnaire

Immediately after the Balance Wood trials, participants completed a structured questionnaire in 4 parts (provided in Multimedia Appendix 4); all items were rated on a 5-point Likert scale. Part 1 is the System Usability Scale (SUS) [48,49], scored at 0 to 100. Part 2 is a set of 9 custom feasibility (use-impression) items in 4 dimensions: feedback comprehension, physical burden and safety, enjoyment and adherence, and perceived ankle specificity of movement. Part 3 asks participants to rate the extent to which they consciously used specific joints, muscles, and body regions during the task (3 rating perspectives; see Tables 2-4); this addresses the concern that the recorded combined tilt signal may reflect contributions from body segments other than the ankle. Part 4 provides space for the free-form description of participant experience, perceived difficulty, and any operational changes noticed compared with prior experience. The total stage 2 testing session lasted approximately 30 minutes per participant.

**Table 2.** Part 3 consciously used joints in stage 2 (n=18, 5-point Likert). CIs extending beyond the 1‐5 rating range were truncated to the scale bounds.

	Mean (SD)	95% CI
Ankle joint	4.94 (0.24)	4.83‐5.00
Toe joints	3.83 (1.20)	3.24‐4.43
Knee joint	2.50 (1.29)	1.86‐3.14
Trunk	2.17 (1.15)	1.59‐2.74
Hip joint	2.00 (1.24)	1.39‐2.61
Shoulder joint	1.11 (0.32)	1.00‐1.27

**Table 3.** Part 3 consciously used muscles in stage 2 (n=18, 5-point Likert). CIs extending beyond the 1‐5 rating range were truncated to the scale bounds.

	Mean (SD)	95% CI
Triceps surae	4.78 (0.73)	4.41‐5.00
Quadriceps femoris	3.28 (1.27)	2.64‐3.91
Hamstrings	2.94 (1.39)	2.25‐3.64
Gluteal muscles	2.56 (1.29)	1.91‐3.20
Trunk muscles	1.94 (1.00)	1.45‐2.44
Upper limb muscles	1.17 (0.51)	1.00‐1.42

**Table 4.** Part 3 consciously used body regions in stage 2 (n=18, 5-point Likert). CIs extending beyond the 1‐5 rating range were truncated to the scale bounds.

	Mean (SD)	95% CI
Ankle region	5.00 (0.00)	5.00‐5.00
Lower leg	4.17 (1.10)	3.62‐4.71
Toes	4.06 (1.21)	3.45‐4.66
Knee region	2.89 (1.41)	2.19‐3.59
Thigh	2.61 (1.38)	1.93‐3.30
Hip region	2.17 (1.20)	1.57‐2.76
Trunk/core	1.83 (0.99)	1.34‐2.32
Upper limb	1.11 (0.32)	1.00‐1.27

#### Stage 2: Outcome Measures

In addition to the 5 signal-based outcome measures analyzed for within-group trial effects in stage 1 (see Outcome measures), stage 2 reported the SUS composite score (0‐100); mean Likert score per part 2 feasibility item and per feasibility dimension; mean Likert ratings of consciously used joints, muscles, and body regions (part 3); and thematic categorization of part 4 open-ended responses.

### Representative Example of a Single-Trial Signal

**Figure 2.**
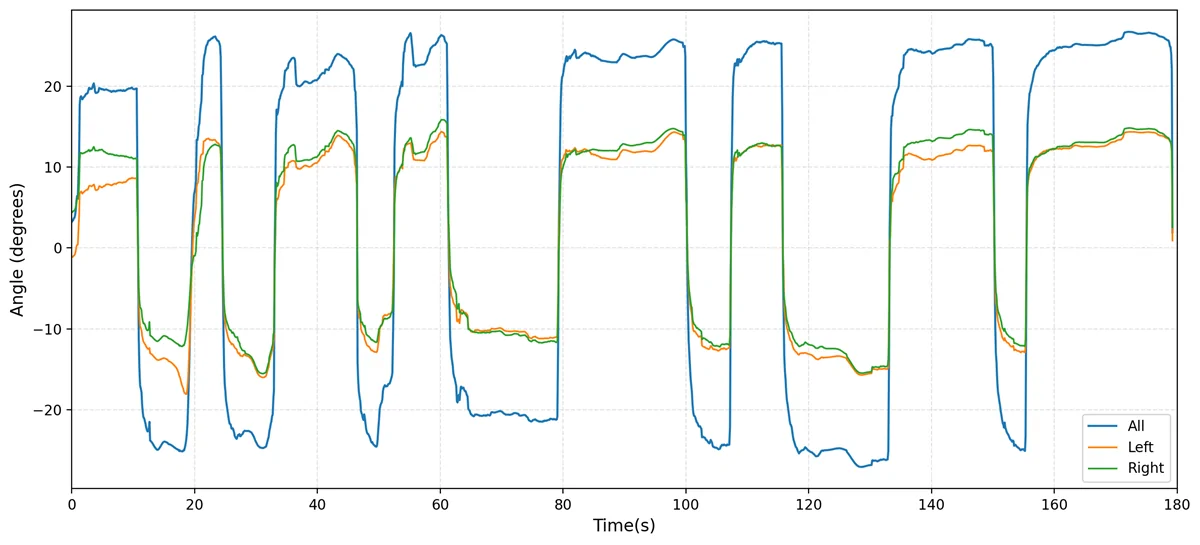
Representative time-series from a single balance wood trial, showing the combined control-angle signal together with the individual left- and right-unit signals. Repeated forward and backward peaks appear throughout this trial; some show a decrease followed by rapid recovery, reflecting adjustment to the adaptive challenge-penalty mechanism. Amplitude and effective flexion-extension analyses used peak segmentation, whereas holding analyses used the filtered effective-trial signal with participant-specific directional near-limit thresholds. Negative values denote forward tilt and positive values denote backward tilt.

A representative signal from trial 3 of a single participant is displayed (Figure 2), covering the full 180-second trial; each peak corresponds to 1 period of maintained forward or backward ankle movement. Some peaks show an amplitude decrease followed by a rapid rise, reflecting adjustment after the challenge-penalty mechanism was triggered. In many peaks, the latter half reached a greater amplitude than the former half, and peaks later in the trial tended to be larger than the initial ones. Such waveforms illustrate the signal features on which the proposed exploratory measures are computed [47].

### Acquisition and Definition of Valid Signals

The parameters used in this study fall into 2 categories. System parameters of the adaptive challenge-penalty mechanism—including the 11° displayed-angle minimum valid peak threshold (equivalent to approximately 18.33° physical tilt), the 0.3° threshold increment, the 95% maintenance criterion, the 7-second observation window, and the 4-second penalty cancellation window—were fixed during system development and constitute part of the experimental environment shared by all participants. These parameters shaped the data-generating process and cannot be retrospectively altered without rerunning the experiment. Offline analysis parameters—including the middle 80% peak retention window, the κ=0.8 coefficient for the directional holding threshold, and the second-half-vs-first-half comparison rule for effective flexion-extension classification—were chosen during signal processing and could in principle be varied to evaluate the robustness of derived measures. This distinction is referenced in the Limitations subsection of the Discussion section.

In this study, valid signal acquisition involved 2 complementary levels of processing. First, direction-specific excursions (peaks) were identified for peak-based analyses such as typical directional amplitude and effective flexion-extension proportion. Second, clearly artifactual or nonrepresentative segments were excluded from the continuous trial signal to obtain an effective analyzed signal for time-based outcomes such as directional holding proportion and overspeed proportion.

For peak-based analyses, a peak was defined as a direction-specific excursion bounded by return toward the neutral level, and analysis focused on the stable portion of each excursion: only the middle 80% of each peak was treated as the valid signal region, the first and last 10% being treated as transition periods between forward and backward movement. A symmetric 10% trim was used as a simple operational rule, retaining most of each excursion while reducing the influence of direction-reversal transitions at both ends.

Clearly artifactual or nonrepresentative segments were also excluded, such as isolated initial transitional segments or extreme abnormal segments whose waveform patterns differed markedly from surrounding peaks. Segment exclusion was performed using predefined rules and verified by visual inspection. This operated within each participant’s recorded data and did not exclude any participant; no participant-level data were missing.

### Outcome Measures

#### Overview

As sensor-based exergame systems can provide information beyond global scores [23,24,43,50], this study prespecified 7 measures in 4 categories, derived from the device tilt-angle signal: typical forward amplitude, typical backward amplitude, forward holding proportion, backward holding proportion, overspeed proportion, forward effective flexion-extension proportion, and backward effective flexion-extension proportion. Of these, 5 (the 2 typical amplitudes, the 2 directional holding proportions, and the overspeed proportion) were analyzed for within-group trial effects in stage 1, whereas the 2 directional effective flexion-extension proportions were pooled across all valid excursions and reported descriptively. These measures are exploratory and descriptive: mathematically defined indicators of device-level ankle-control behavior that are not used to assess functional ability, clinical status, or training effects, and whose definition does not establish biomechanical validity.

#### Typical Flexion-Extension Amplitude

Typical flexion-extension amplitude quantifies the typical displacement produced by ankle flexion-extension in a given direction rather than the single most extreme value. It was calculated separately for forward tilt (negative values) and backward tilt (positive values). For each valid excursion, including both valid forward and valid backward excursions, the maximum angular magnitude in the corresponding direction was extracted. The amplitudes of all valid excursions within a trial were then averaged.

Let $N_{exc}(k)$ denote the total number of valid excursions in trial k, and let $a_{i}(k)$ denote the amplitude of the i-th valid excursion, expressed as an absolute value. Typical flexion-extension amplitude was defined as


$$A_{flex}(k)=\frac{1}{N_{exc}(k)}\sum_{i=1}^{N_{exc}(k)}a_{i}(k).$$

Averaging across excursions makes this measure less sensitive to isolated outlier peaks than a single-trial maximum.

#### Directional Holding Proportion

Directional holding proportion quantified the proportion of effective trial time during which the participant maintained the control angle within a near-limit region specific to the participant in a given direction. Unlike a peak-count-based definition, this measure was defined as a time proportion over the full analyzed signal after the exclusion of invalid segments.

To improve within-participant comparability across trials, the near-limit threshold for each direction was defined from the typical directional amplitude of the participant in trial 2. Trial 2 was selected as this reference because the easy condition was typically completed too early and the high-difficulty condition could not be completed at all, whereas the medium-difficulty condition was preset to be just completable within the time limit and therefore provided the most representative and stable estimate of each participant’s typical directional amplitude. This anchoring was adopted as an early, exploratory choice rather than a validated standard. Specifically, for backward tilt (positive values), the threshold was defined as


$$T_{backward}=\kappa A_{backward}(2),$$

and for forward tilt (negative values), the threshold was defined as


$$T_{forward}=-\kappa A_{forward}(2),$$

where κ=0.8. Thus, the holding region was defined as tilt at or beyond 80% of the participant-specific typical directional amplitude obtained from trial 2. The value κ=0.8 was used as an operational near-limit threshold, allowing sustained high-amplitude tilt to be captured without restricting the measure to only the brief extreme peak. The same coefficient was applied consistently across participants and trials.

Let $T_{eff}(k)$ denote the total effective analyzed duration in trial k after the exclusion of invalid segments. For backward tilt, let $T_{hold,backward}(k)$ denote the total duration during which the filtered tilt-angle signal of the device remained at or above $T_{backward}$. For forward tilt, let $T_{hold,forward}(k)$ denote the total duration during which the filtered tilt-angle signal of the device remained at or below $T_{forward}$. The directional holding proportions were defined as


$$H_{backward}(k)=\frac{T_{hold,backward}(k)}{T_{eff}(k)},$$

$$H_{forward}(k)=\frac{T_{hold,forward}(k)}{T_{eff}(k)}.$$

In addition, for descriptive presentation, a combined holding proportion can be calculated as


$$H_{combined}(k)=H_{forward}(k)+H_{backward}(k)=\frac{T_{hold,forward}(k)+T_{hold,backward}(k)}{T_{eff}(k)},$$

which represents the proportion of effective trial time spent in either directional near-limit region; the forward and backward proportions were retained separately in the main analysis.

#### Directional Effective Flexion-Extension Proportion

Directional effective flexion-extension proportion was calculated separately for forward and backward movements to evaluate whether participants demonstrated continued amplitude increase within the valid signal segment. For each valid excursion, that is, the middle 80% of the peak, only the high-amplitude top region was retained. The steep entering and leaving edges of this top region were then trimmed on the basis of local slope, leaving a window intended to represent the plateau-like upper portion of the excursion while excluding rapid rising and falling transitions.

This top window was then divided into a first half and a second half, and the mean amplitude of each half was calculated. If the mean amplitude of the second half was greater than that of the first half, the excursion was classified as an effective excursion. Intuitively, this indicates that during the valid, stable phase of flexion-extension, the participant continued to increase or at least sustain high amplitude rather than allowing the movement to decline.

Let $N_{top}(k)$ denote the number of valid excursions analyzed in trial k, and let $N_{eff}(k)$ denote the number of excursions satisfying the criterion that the latter-half amplitude exceeded the former-half amplitude. Effective flexion-extension proportion was defined as


$$E(k)=\frac{N_{eff}(k)}{N_{top}(k)}.$$

When an all-3-trials combined value is reported, it is calculated by pooling all valid excursions across trials 1, 2, and 3 and then recomputing $E\text{=}N_{eff}\text{/}N_{top}$ rather than by averaging the 3 trial-level proportions. Higher values indicate that a larger proportion of valid excursions sustained or increased amplitude during the latter part of the peak.

#### Overspeed Proportion (Additional Outcome)

Overspeed proportion was included as an additional analysis item and was not treated as a core ankle flexion-extension outcome. The main purpose of this outcome was to examine the influence of the mechanism of overspeed leakage on the behavior of the participant. Specifically, it quantifies the proportion of analyzed time samples in which angular velocity exceeded a preset threshold. This angular-velocity threshold is a fixed system parameter of the speed-feedback (water-spill) mechanism and is constant across difficulty levels.

Let v(t) denote the instantaneous angular velocity, $v_{thr}$ the preset physical angular-velocity threshold used in the final analysis pipeline, $N_{all}(k)$ the total number of analyzed samples in trial k, and $N_{\text{over}}(k)$ the number of samples for which $\left|v_{t}\right|§gt;v_{thr}$. Overspeed proportion was defined as


$$O(k)=\frac{N_{over}(k)}{N_{all}(k)}.$$

As the threshold is a fixed feature of the speed-feedback mechanism, this proportion is not interpreted as a measure of motor control.

### Statistical Analysis

#### Stage 1: Statistical Analysis

All stage 1 analyses were conducted separately for the older-adult and younger-adult groups. No between-group inferential tests were performed and no between-group comparisons are reported, because this study was neither designed nor powered for between-group comparison; the 2 stage 1 groups are described separately. No data were missing in stage 1.

For the 5 main trial-level outcomes, descriptive statistics were calculated for each trial. To evaluate within-group trial effects across trials 1, 2, and 3, a 1-factor repeated-measures ANOVA was performed for each outcome. Sphericity was assessed using the Mauchly test, with Greenhouse-Geisser correction applied when violated, and normality using the Shapiro-Wilk test. For each ANOVA, we report *F* test scores, df1, df2, *P* values, and partial eta squared, with 95% CIs for post hoc pairwise mean differences. As 5 outcomes were tested per group, outcome-level multiplicity was addressed using a Bonferroni-corrected threshold of *P*<.01, with uncorrected P values retained. Supplementary Friedman tests were conducted for outcomes showing deviations from normality. Effect sizes were interpreted against conventional benchmarks (ηp²=0.01, 0.06, and 0.14 for small, medium, and large).

#### Stage 2: Statistical Analysis

Difficulty and presentation-order effects. For each signal-based measure, differences across the 3 difficulty conditions and, separately, across the 3 presentation positions were examined using repeated-measures ANOVA. As the design was fully counterbalanced, difficulty was decoupled from presentation order; with 3 per ordering, ordering-specific summaries are descriptive only.

Regarding usability and feasibility, the SUS composite score was reported as the mean (SD) with a 95% CI and compared with the conventional 68-point benchmark; internal consistency was summarized using Cronbach α. Part 2 and 3 ratings were reported as means (SDs) with 95% CIs, and part 3 ankle ratings were compared with proximal-segment ratings using Wilcoxon signed-rank tests. Part 4 responses were analyzed using inductive content analysis, with 2 coders independently coding the responses and grouping them into themes through discussion. There were no missing item-level data in the stage 2 questionnaire, and no imputation was required.

#### Software

All statistical analyses were performed using custom scripts written in Python (version 3.10; Python Software Foundation), with pandas, NumPy, SciPy (version 1.15.3), and Matplotlib (version 3.10.8).

## Results

### Overview

**Figure 3.**
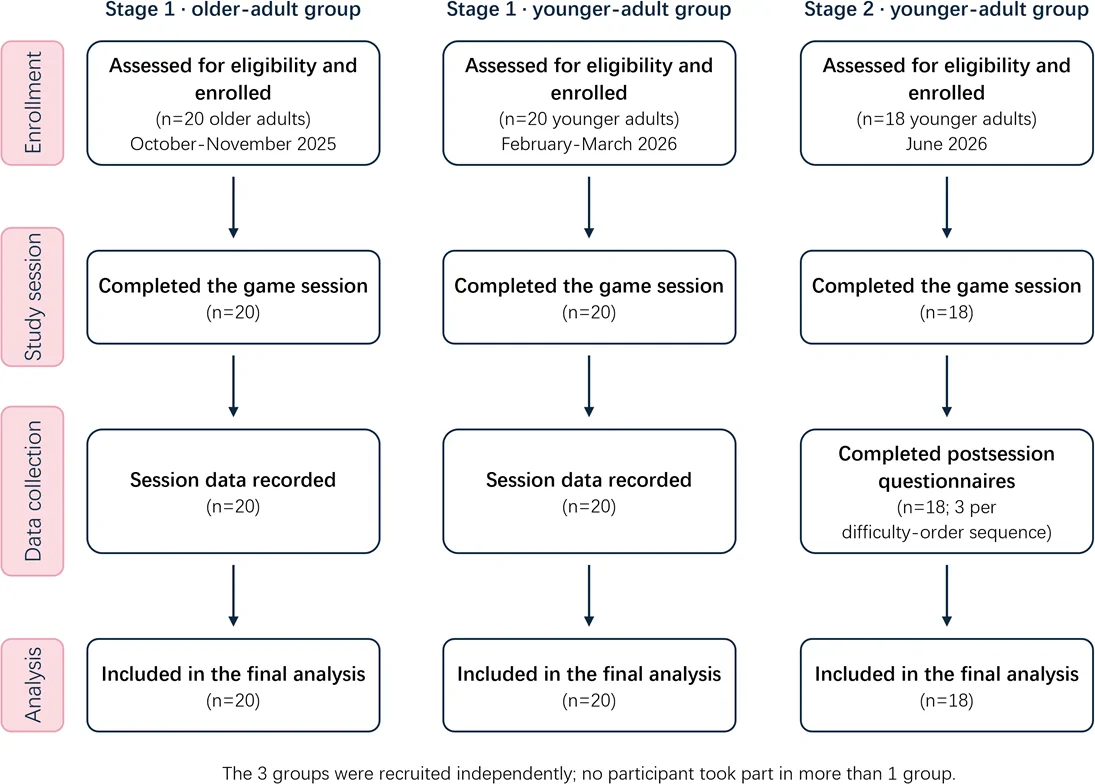
Participant flowchart across the 2 study stages, following the American Psychological Association Journal Article Reporting Standards for quantitative research. The 3 groups (stage 1 older adults, stage 1 younger adults, and stage 2 younger adults) were recruited independently—the older adults from the general community with support from the JST (Japan Science and Technology Agency) Moonshot program (JPMJMS2034), and the younger adults by convenience sampling from the Tokyo Metropolitan University community—and all were assessed at the Graduate School of Systems Design, Tokyo Metropolitan University (Hino campus, Tokyo, Japan); no participant took part in more than one group. All enrolled participants completed this study’s procedures, with no exclusions or withdrawals.

All 58 enrolled participants (stage 1: 20 older and 20 younger adults; stage 2: 18 younger adults) completed all study procedures and were included in the analysis, with no exclusions or withdrawals (the flow of participants through the 3 groups is shown in Figure 3). In stage 2, all 18 participants provided complete questionnaire responses, with no missing data for the usability outcomes.

### Older-Adult Group

The descriptive statistics of the main outcomes in the older-adult group are summarized (Table 5). When all 3 trials were combined by pooling valid excursions across the 3 trials, the effective flexion-extension proportion was 0.838 in the forward direction and 0.742 in the backward direction.

**Table 5.** Descriptive statistics of the main outcomes in the older-adult group (n=20). Amplitude values are reported on the true physical angle scale. Combined holding proportion was included for descriptive presentation only. Where a per-participant SD was available, 95% CIs (mean ± t(19) × SD/√20) are shown in parentheses; the combined and effective flexion-extension proportions are aggregate ratios with no per-participant SD and are therefore reported as point values only.

		Trial 1	Trial 2	Trial 3
Typical forward amplitude (deg)				
Mean (SD)		21.490 (2.609)	23.204 (4.255)	23.312 (3.260)
95% CI		20.27 to 22.71	21.21 to 25.20	21.79 to 24.84
Median (IQR)		21.763 (19.970-23.047)	23.336 (21.209-25.815)	23.776 (21.042-25.747)
Typical backward amplitude (deg)				
Mean (SD)		20.478 (3.176)	22.302 (1.777)	23.050 (4.839)
95% CI		18.99 to 21.96	21.47 to 23.13	20.79 to 25.31
Median (IQR)		20.358 (18.319-21.980)	21.985 (21.118-23.702)	22.016 (21.229-23.561)
Forward holding proportion				
Mean (SD)		0.324 (0.152)	0.383 (0.130)	0.436 (0.135)
95% CI		0.253 to 0.395	0.322 to 0.444	0.373 to 0.499
Median (IQR)		0.352 (0.230-0.416)	0.394 (0.305-0.496)	0.439 (0.352-0.539)
Backward holding proportion				
Mean (SD)		0.392 (0.147)	0.437 (0.117)	0.353 (0.139)
95% CI		0.323 to 0.461	0.382 to 0.492	0.288 to 0.418
Median (IQR)		0.434 (0.303-0.485)	0.434 (0.355-0.492)	0.326 (0.313-0.435)
Combined holding proportion				
Proportion		0.716	0.820	0.789
Effective flexion-extension proportion (forward)				
Proportion		0.785	0.860	0.848
Effective flexion-extension proportion (backward)				
Proportion		0.536	0.816	0.799
Overspeed proportion				
Mean (SD)		0.051 (0.018)	0.048 (0.016)	0.051 (0.013)
95% CI		0.043 to 0.059	0.041 to 0.055	0.045 to 0.057
Median (IQR)		0.051 (0.037-0.060)	0.045 (0.041-0.056)	0.051 (0.044-0.057)

The results of the Mauchly test are reported for all 5 outcomes (Table S2 in Multimedia Appendix 3). The sphericity assumption was violated only for the typical backward amplitude $\left(W=0.5432,\;P=.0041,\;\epsilon_{GG}=0.686\right)$, therefore, Greenhouse-Geisser corrected results were reported for that outcome. The repeated-measures ANOVA revealed nominally significant trial effects for the typical forward amplitude (F₂,₃₈=4.169, P=.023, ηp²=.180), and the typical backward amplitude (F₁.₃₇₃,₂₆.₀₈₄=4.338, P=.036, ηp²=.186). The forward holding proportion ($F_{2,38}\text{=}3.122$, P=.056, $\eta_{p}^{2}\text{=}.141$) and the backward holding proportion ($F_{2,38}\text{=}2.563$, P=.090, $\eta_{p}^{2}\text{=}.119$) did not reach nominal significance. The overspeed proportion remained stable across the 3 trials ($F_{2,38}\text{=}0.416$, P=.663, $\eta_{p}^{2}\text{=}.021$). Under the Bonferroni-corrected threshold of P§lt;.01, neither amplitude effect reached formal significance, and both should be read as preliminary despite their large effect sizes. Post hoc paired comparisons with Bonferroni correction were conducted for the 2 outcomes demonstrating trial effects. For the typical forward amplitude, no pairwise comparison remained significant after correction, although increases from trial 1 to trials 2 and 3 were observed descriptively. By contrast, for the typical backward amplitude, the increase from trial 1 to trial 2 remained significant after Bonferroni correction ($t_{19}\text{=}3.518$, P=.0023, $P_{adj}\text{=}.0069$, $d_{z}\text{=}0.787$). The increase from trial 1 to trial 3 was additionally retained as a meaningful descriptive change, although it did not remain significant after correction (Table S3 in Multimedia Appendix 3).

Shapiro-Wilk testing indicated approximately normal trial-level distributions apart from the typical backward amplitude in trial 3. A supplementary Friedman test was therefore conducted for that outcome as a sensitivity analysis. It was additionally significant ($\chi_{2}^{2}\text{=}9.30$, P=.0096, Kendall W=0.233; Table S4 in Multimedia Appendix 3) and did not change the interpretation of the repeated-measures ANOVA result.

Overall, typical amplitudes were higher in the later trials than in trial 1, the clearest pairwise change being the increase in typical backward amplitude from trial 1 to trial 2. The forward holding proportion increased gradually across trials, the backward holding proportion peaked in trial 2 before declining in trial 3, and the overspeed proportion remained stable. The effective flexion-extension proportion suggested a direction-dependent movement pattern rather than a uniform change across both directions.

### Younger-Adult Group

The descriptive statistics of the main outcomes in the younger-adult group are summarized (Table 6). When all 3 trials were combined by pooling valid excursions across the 3 trials, the effective flexion-extension proportion was 0.779 in the forward direction and 0.787 in the backward direction.

**Table 6.** Descriptive statistics of the main outcomes in the younger-adult group (n=20). Amplitude values are reported on the true physical angle scale. Combined holding proportion was included for descriptive presentation only. Where a per-participant SD was available, 95% CIs (mean ± t(19) × SD/√20) are shown in parentheses; the combined and effective flexion-extension proportions are aggregate ratios with no per-participant SD and are therefore reported as point values only.

	Trial 1	Trial 2	Trial 3
Typical forward amplitude (deg)			
Mean (SD)	23.464 (3.870)	24.990 (3.823)	24.140 (4.538)
95% CI	21.65 to 25.28	23.20 to 26.78	22.02 to 26.26
Median (IQR)	23.858 (22.178-25.523)	24.277 (22.698-26.600)	23.324 (21.336-25.804)
Typical backward amplitude (deg)			
Mean (SD)	26.063 (3.861)	26.755 (2.813)	27.213 (3.258)
95% CI	24.26 to 27.87	25.44 to 28.07	25.69 to 28.74
Median (IQR)	26.119 (24.410-28.454)	26.268 (24.868-28.516)	27.488 (25.591-29.142)
Forward holding proportion			
Mean (SD)	0.313 (0.154)	0.400 (0.130)	0.383 (0.160)
95% CI	0.241 to 0.385	0.339 to 0.461	0.308 to 0.458
Median (IQR)	0.304 (0.231-0.428)	0.403 (0.312-0.490)	0.447 (0.264-0.496)
Backward holding proportion			
Mean (SD)	0.449 (0.201)	0.433 (0.126)	0.414 (0.159)
95% CI	0.355 to 0.543	0.374 to 0.492	0.340 to 0.488
Median (IQR)	0.411 (0.366-0.532)	0.408 (0.364-0.518)	0.381 (0.310-0.479)
Combined holding proportion			
Proportion	0.762	0.833	0.797
Effective flexion-extension proportion (forward)			
Proportion	0.769	0.798	0.836
Effective flexion-extension proportion (backward)			
Proportion	0.621	0.783	0.810
Overspeed proportion			
Mean (SD)	0.049 (0.017)	0.050 (0.012)	0.049 (0.010)
95% CI	0.041 to 0.057	0.044 to 0.056	0.044 to 0.054
Median (IQR)	0.043 (0.037-0.065)	0.049 (0.042-0.057)	0.048 (0.041-0.058)

The results of the Mauchly test are reported for all 5 outcomes (Table S5 in Multimedia Appendix 3). The sphericity assumption was violated only for the typical forward amplitude; therefore, Greenhouse-Geisser corrected results were reported for that outcome. The repeated-measures ANOVA showed no significant trial effect for any of the 5 main inferential outcomes in the younger-adult group, with effect sizes that were small to medium at most (partial ηp²=0.083 or below). Accordingly, no formal post hoc paired comparisons were performed for the younger-adult group.

Shapiro-Wilk testing indicated that most trial-level distributions in the younger-adult group were approximately normal, although deviations from normality were observed for the typical forward amplitude in all 3 trials and for the overspeed proportion in trial 3. Therefore, supplementary Friedman tests were conducted for these outcomes as sensitivity analyses. These tests were not significant for the typical forward amplitude ($\chi_{2}^{2}\text{=}1.90$, P=.3867, Kendall W=0.048) or the overspeed proportion ($\chi_{2}^{2}\text{=}1.30$, P=.5220, Kendall W=0.033; Table S4 in Multimedia Appendix 3) and thus did not change the overall interpretation of the repeated-measures ANOVA results. Overall, the younger-adult group showed minimal within-session trial-wise change: typical amplitudes, holding proportions, and the overspeed proportion all remained relatively stable across the 3 trials, and the forward and backward effective flexion-extension proportions were similar to one another.

### Stage 2 Results: Counterbalanced Supplementary Study

#### Overview

Consistent with the development-and-usability framing, the usability and feasibility findings are the primary stage 2 outcomes; the signal-derived analyses are reported as secondary, exploratory observations.

#### Stage 2: Participant Characteristics

Stage 2 enrolled 18 younger adults (mean age 24.6, SD 3.9 y; 5 women), recruited by convenience sampling from the research laboratory community. The 3 difficulty conditions were administered in a fully counterbalanced order: the 6 possible orderings of the easy, medium, and hard conditions (denoted L, M, and H, respectively: LMH, LHM, MLH, MHL, HLM, and HML) were each assigned to 3 participants, yielding a fully balanced Latin square in which each difficulty condition appeared equally often in each presentation position. As participants were drawn from the laboratory community in which Balance Wood was developed, they differed in their degree of prior exposure to the system. Detailed demographic characteristics are summarized in Table 1 alongside the stage 1 participants.

#### Perceived Usability (SUS)

The mean SUS composite score was 81.5 (SD 11.2; 95% CI 76.0‐87.1; median 83.75, IQR 78.13-87.50; range 47.5‐95.0; Cronbach *α*=0.74, indicating acceptable internal consistency). This score is well above the established benchmark of 68, indicating good perceived usability, although individual variability was present (minimum 47.5). Item-level results are summarized in Table 7: positively worded items received high ratings, and negatively worded items received low ratings, consistent with good usability, and the lowest-scoring positively worded item was Q1 (“I think that I would like to use this system frequently”).

**Table 7.** SUS^a^ item-level scores in stage 2 (n=18). Odd-numbered items are positively worded, and even-numbered items are negatively worded; values are mean (SD) with 95% CIs on the 1‐5 response scale. The overall SUS score (0‐100) was 81.5 (95% CI 76.0‐87.1).

	Mean (SD)	95% CI
Q1 (+) would like to use frequently	3.33 (0.84)	2.92‐3.75
Q2 (−) unnecessarily complex	1.83 (0.92)	1.37‐2.29
Q3 (+) easy to use	4.67 (0.59)	4.37‐4.96
Q4 (−) need help of a knowledgeable person	1.89 (0.83)	1.47‐2.30
Q5 (+) functions are well integrated	4.11 (0.83)	3.70‐4.53
Q6 (−) too much inconsistency	1.72 (0.96)	1.25‐2.20
Q7 (+) most people would learn quickly	4.61 (0.61)	4.31‐4.91
Q8 (−) cumbersome to operate	1.44 (0.78)	1.05‐1.83
Q9 (+) felt confident	4.33 (0.69)	3.99‐4.67
Q10 (−) needed to learn a lot first	1.56 (0.98)	1.07‐2.04

a SUS: System Usability Scale.

#### Use Impressions

Item-level results for the 9 part 2 use-impression items are summarized in Table 8. The 2 feedback-comprehension items received high ratings; safety concerns and pain or discomfort were rated low, and participants rated using mainly ankle movement higher than also using nonankle body parts. As part 2 contains both positively and negatively worded items, the items are reported individually rather than as a single composite.

**Table 8.** Part 2 use-impression item scores in stage 2 (n=18, 5-point Likert).

	Mean (SD)	95% CI
1. Visual feedback helped me understand the task purpose	4.39 (0.61)	4.09‐4.69
2. Spill-on-fast-switch feedback helped me adjust speed	4.22 (0.65)	3.90‐4.54
3. Felt ankle fatigue over the 3 trials	3.61 (1.33)	2.95‐4.27
4. Concerned about safety during operation	2.11 (1.32)	1.45‐2.77
5. Experienced pain or discomfort	1.67 (1.03)	1.15‐2.18
6. Found the game enjoyable	3.78 (0.94)	3.31‐4.25
7. Willing to use regularly (2‐3×/wk)	3.61 (0.85)	3.19‐4.03
8. Mainly used ankle movement	4.28 (0.96)	3.80‐4.75
9. Additionally used nonankle body parts	2.94 (1.43)	2.23‐3.66

#### Consciously Used Body Parts

Part 3 asked participants to rate the extent to which they consciously used specific joints, muscles, and body regions, on 3 rating perspectives (Tables 2-4). Across all 3 perspectives, the ankle and adjacent structures (lower leg and toes) received the highest ratings, and proximal segments received progressively lower ratings. For the joints perspective, Wilcoxon signed-rank tests comparing the ankle joint with the knee, hip, and trunk were all statistically significant (*P*<.001). This pattern is reported descriptively and is not interpreted as establishing the absence of compensatory movement.

#### Qualitative Feedback

Twelve of the 18 participants provided free-text responses, which were analyzed using inductive content analysis. Participants generally found the task realistic and engaging and reported clearly feeling the activity at the ankle, which was noted as a strength. The most frequently raised area for improvement was foot slippage: several participants reported that, with some types of footwear, the foot tended to slip or leave the board and requested a fixation or strap. Additional comments noted ankle fatigue at the highest difficulty level and offered suggestions regarding the intuitiveness of the displayed tilt direction.

#### Device Tilt-Angle Accuracy

To characterize the accuracy of the device tilt-angle output, a bench-level standard-angle test was performed. The Balance Wood platform was tilted to 5 reference angles (0°, 10°, 20°, 30°, and 40°) in both the forward and backward directions, for each of the 2 units, with 3 repetitions per condition (60 measurements in total). At each static position, the reference angle was read with a digital goniometer (SCITOOLS, China; display resolution 0.01°) while the corresponding device tilt-angle value transmitted over User Datagram Protocol was recorded. Agreement was quantified using the metrics listed in Table 9, including the mean absolute error (MAE), root-mean-square error, and the intraclass correlation coefficient (ICC; 2-way mixed, absolute agreement). This is a device-level test of physical tilt-angle measurement and does not assess the anatomical ankle joint angle.

The device output showed close agreement with the goniometer reference, with an MAE of 0.66° (root-mean-square error 0.79°), excellent linearity (*R*²=0.998), and excellent agreement (ICC=0.999; Bland-Altman 95% limits of agreement −0.68° to 1.70°). A small systematic bias of +0.51° was observed, and within the operating range observed during gameplay (20°-30°), the MAE was 0.73°. Subdegree agreement is expected when rigid device tilt rather than a human joint angle is measured; joint-angle measurement typically yields errors of several degrees [31,32,43]. Accuracy metrics and per-angle results are summarized in Tables 9 and 10.

**Table 9.** Device tilt-angle accuracy against a digital goniometer (SCITOOLS; range 0°-360°, display resolution 0.01°), standard-angle bench test (5 reference angles × 2 directions × 2 units × 3 repetitions = 60 measurements). The test characterizes the physical device tilt angle, not the anatomical ankle joint angle.

	Value
Mean absolute error	0.66°
Root-mean-square error	0.79°
Systematic bias	+0.51°
Intraclass correlation coefficient	0.999
Linear regression (*R***²**)	0.998
Within-condition SD (repeatability)	0.53°
MAE^a^ within operating range (20°-30**°**)	0.73°
Bland-Altman 95% limits of agreement	−0.68° to 1.70°

a MAE: mean absolute error.

**Table 10.** Per-angle device tilt-angle accuracy against the digital goniometer (SCITOOLS), standard-angle bench test (n=12 per reference angle: 2 directions × 2 units × 3 repetitions).

	MAE^a^	Bias	Maximum absolute error	n
Reference angle 0°	0.77°	+0.65°	1.80°	12
Reference angle 10°	0.48°	+0.20°	1.00°	12
Reference angle 20°	0.76°	+0.46°	1.40°	12
Reference angle 30°	0.70°	+0.65°	1.50°	12
Reference angle 40°	0.59°	+0.59°	1.30°	12

a MAE: mean absolute error.

#### Counterbalanced Signal-Derived Measures (Additional Exploratory Analysis)

As an additional exploratory analysis, the 7 signal-derived measures defined in stage 1 were recomputed per participant for the stage 2 sample under the counterbalanced design (the combined holding proportion, a derived aggregate, was not included). Differences across the 3 difficulty conditions and across presentation positions were examined using repeated-measures ANOVA. No statistically significant differences were observed across difficulty conditions for any measure (typical forward amplitude: easy 24.8°, 95% CI 23.0‐26.5; medium 24.2°, 95% CI 23.1‐25.4; hard 24.0°, 95% CI 22.9‐25.2; *F*₂,₃₄=0.75, *P*=.48; typical backward amplitude *F*₂,₃₄=1.41, *P*=.26; forward holding proportion *F*₂,₃₄=1.31, *P*=.28; backward holding proportion *F*₂,₃₄=1.67, *P*=.20; forward effective flexion-extension proportion *F*₂,₃₄=0.76, *P*=.48; backward effective flexion-extension proportion *F*₂,₃₄=0.41, *P*=.67; overspeed proportion *F*₂,₃₄=1.08, *P*=.35), and no significant effects of presentation position were observed (all *P*>.13; the 2 amplitude measures, *P*=.73 and *P*=.30). The complete output for both factors is given in Table S6 in Multimedia Appendix 3. These analyses are exploratory, and no inference regarding responsiveness to task difficulty is drawn.

## Discussion

### Principal Results

This study developed Balance Wood, a bilateral ankle-controlled serious game, and evaluated its usability and the kinds of gameplay-derived signals it records in healthy adults. Across both stages, the system produced continuous tilt-angle signals from which the proposed exploratory measures could be computed.

In stage 1, the proposed measures could be computed for every participant, and each group is described separately. Within the older-adult group, typical directional amplitudes increased across the 3 trials; within the younger-adult group, the measures remained comparatively stable. The overspeed proportion varied little across trials in both groups. Taken separately, they illustrate how continuous signal tracking can characterize movement patterns beyond simple task completion.

In stage 2, under a fully counterbalanced administration in younger adults, the signal-derived measures showed no significant differences across difficulty conditions or presentation positions, and the device tilt angle agreed closely with a digital goniometer at the device level. On the structured questionnaire, perceived usability was good, safety concerns and discomfort were rated low, and participants reported using predominantly the ankle and adjacent structures rather than proximal segments.

Taken together, the 2 stages provide complementary evidence for the signal-derived framework. Stage 1 showed that the measures could be extracted from repeated gameplay and describe within-session patterns. Stage 2 extended the framework to an independent, counterbalanced younger-adult sample, in which all 7 measures were again derived and no statistically significant effects of difficulty or presentation position were detected. The methodological contribution of stage 2 is therefore that the framework remains applicable when difficulty and presentation position are separated by design. Accordingly, stage 1 patterns are best interpreted as descriptions of repeated gameplay rather than effects attributable specifically to task difficulty or order. Stage 2 findings remain exploratory because of the small, younger-adult-only sample and heterogeneous prior exposure.

### Comparison With Prior Work

Previous exergame studies and reviews have evaluated usability and acceptability across younger and older adult populations [10,13,20,42]. As usability depends on interaction design and use context, findings from other exergame configurations cannot be assumed to transfer directly to Balance Wood’s seated, bilateral, ankle-focused interface. In stage 2, the mean SUS score was 81.5, above the conventional benchmark of 68 [49] and descriptively higher than the score of 70.5 reported for a home-based exergame evaluated in a similarly aged healthy sample [10]. Participants also reported that visual feedback helped them understand the task and adjust their speed, rated safety concern and pain or discomfort low, and indicated predominant reliance on the ankle and adjacent structures. Ratings for enjoyment and willingness to use regularly were 3.78 and 3.61, respectively, on a 5-point scale. These comparisons remain descriptive given the small, younger-adult stage 2 sample and heterogeneous prior exposure to the system.

Controlled laboratory work has characterized bilateral ankle-dorsiflexion force control using customized force-measurement platforms and target-matching tasks [3]. Although informative, this approach was not designed as a serious game and relied on a laboratory-oriented setup. Exergame studies have also quantified movement using 3D motion capture [51] or force-platform center-of-pressure data [52]. Balance Wood instead integrates sensing, visual feedback, and continuous signal recording in a low-cost, seated serious-game interface, deriving direction-specific measures directly from its own bilateral tilt-control signal. Following further validation, this design may facilitate repeated recording in home or community settings.

Finally, prior work has used self-adaptive serious games to adjust task demands during motor learning [53], while related exergame research has examined therapeutic ankle outcomes [54]. In Balance Wood, however, adaptation served a different methodological purpose: rather than primarily targeting motor-learning or therapeutic outcomes, the challenge-penalty mechanism progressively updated the direction-specific peak criterion to encourage repeated, sustained excursions near each participant’s current peak, supporting the capture of high-amplitude movement for descriptive signal analysis.

### Implications for Research and Practice

Balance Wood provides a compact, relatively low-cost method for collecting continuous bilateral ankle-controlled gameplay signals in 1 session; stage 2 required approximately 30 minutes per participant and no laboratory-grade motion-capture equipment for device-level tilt recording. The platform can support standardized signal acquisition across repeated trials alongside end point outcomes. Favorable usability and feedback comprehension, with low discomfort and safety concerns, support further evaluation in applied settings. Following external validation, the system could be evaluated in community settings, small clinics, or home-based research, potentially lowering the logistical barriers to repeated recording of ankle-controlled behavior. At present, Balance Wood is a research and development platform rather than a clinical assessment or diagnostic instrument. Before independent or unsupervised deployment, future studies should examine biomechanical validity, between-session test-retest reliability, usability, and signal characteristics in older and clinical populations, footwear-related slippage, device fixation, and safety procedures.

### Limitations

First, the samples were small (20 per group in stage 1; 18 in stage 2), and several outcomes did not survive multiplicity correction; the results are therefore presented as exploratory and should be read through effect sizes and descriptive patterns rather than *P* values alone. Second, generalizability is limited by the single-site, healthy, and partly younger-adult samples (stage 2 younger adults only, predominantly male, recruited from the university), and between-session test-retest reliability was not assessed. Validation in older and clinical populations is planned as future work.

Third, the signal-derived measures are exploratory descriptors, not validated biomechanical constructs, and may be specific to the Balance Wood task. As the adaptive challenge-penalty mechanism was designed to shape gameplay, its influence on the recorded signals cannot be separated retrospectively; establishing the biomechanical meaning of the measures and systematically varying the mechanism’s parameters are left for future work. The near-limit holding threshold was anchored to each participant’s own trial 2 amplitude, so the resulting holding proportions should be read as relative to each participant’s medium-difficulty performance rather than against an external standard. Fourth, no concurrent validation against gold-standard anatomical measures (eg, goniometry) was performed, so the measures characterize device-level rather than anatomical ankle control; mapping device tilt to the anatomical ankle angle remains for future work.

### Conclusions

This study introduced Balance Wood, a low-cost bilateral serious game that converts ankle-controlled device tilt into continuous gameplay signals, and reported its first development-and-usability evaluation in healthy adults. The system showed good perceived usability (above the established SUS benchmark), a positive and low-risk experience, close agreement between its device-level tilt readings and a digital goniometer, and predominantly ankle-based control. Its novelty lies not in gamification itself but in packaging a low-cost, bilateral, ankle-specific control interface with continuous signal capture into a single, easy-to-use device, which distinguishes it from existing systems that record continuous movement data and are typically laboratory-grade, costly, or oriented toward whole-body or upper-limb movement. Its principal contribution is a reproducible, usability-tested platform with characterized device-level accuracy and formally defined candidate measures. These measures require clinical or biomechanical validation before home- or community-based research; in the near term, the system remains a research tool.

## Supplementary material

10.2196/96617 Multimedia Appendix 1 Video of the standard screen-based version of Balance Wood during task performance.

10.2196/96617 Multimedia Appendix 2 Video of the low-cost LED version of Balance Wood during task performance.

10.2196/96617 Multimedia Appendix 3 Detailed hardware and signal-acquisition specifications, the displayed-vs-physical tilt-angle explanation, the complete adaptive challenge-penalty rules and parameter table, and the stages 1 and 2 repeated-measures ANOVA, post hoc comparison, and sensitivity-analysis tables.

10.2196/96617 Multimedia Appendix 4 The posttask questionnaire administered in stage 2, in English and Japanese.

## Abbreviations

ICC: intraclass correlation coefficient
JST: Japan Science and Technology Agency
MAE: mean absolute error
SUS: System Usability Scale
