# Supplementary material for: Balance Wood, a Bilateral Ankle-Controlled Serious Game for Healthy Adults: 2-Stage Cross-Sectional Development and Usability Study
Source: JMIR Serious Games. 2026 Sep 25;14:e96617. doi: 10.2196/96617 (PMC13614508; doi:10.2196/96617)
Supplement: Supplementary file 1 [file games-v14-e96617-s003.pdf]

Supplementary Methods and Statistical Tables

Hardware and Signal-Acquisition Specifications

The embedded inertial measurement unit is an MPU6886 (integrated in the M5StickC PLUS), a six-axis sensor (three-axis accelerometer and three-axis gyroscope) with 16-bit analog-to-digital conversion. The transmitted tilt value is the roll component of the attitude estimate produced by the module's on-board sensor-fusion routine, which combines the accelerometer and gyroscope signals. According to the manufacturer specification, the accelerometer noise density is 100 μg/√Hz and the gyroscope noise density is approximately ±4 mdps/√Hz. As is typical of low-cost micro-electro-mechanical-systems (MEMS) inertial sensors, the module exhibits a fixed mounting and zero-point offset (approximately −10° when the device rests flat), which arises from the installation angle of the sensor within the device and the inherent zero bias of the sensor and cannot be fully eliminated at the factory stage. This offset was removed before each session by an initial alignment (zeroing) performed with the device in the flat, neutral position, so that all recorded signals were referenced to 0°. Signals were sampled at approximately 9.5 Hz.

Displayed Versus Physical Tilt Angle

The recorded tilt-angle signal from each Balance Wood unit corresponds to the physical inclination of the foot device as measured directly by the M5StickC PLUS module. For game display purposes only, the host system applies a scaling factor of 3/5 to map the physical angle to the visual representation of the bucket on the screen. This visual-display scaling was introduced during system development to keep the on-screen bucket tilt within a perceptually appropriate range for the water-flow physical simulation: at physical inclinations producing displayed angles substantially below the operational threshold, the bucket fails to engage water collection; at physical inclinations producing displayed angles substantially above the threshold, water spills before the bucket can fill. The 3/5 ratio was selected to balance these constraints during pilot testing. A displayed angle of 11° therefore corresponds to approximately 18.33° of physical device tilt.

This scaling factor affects only the visual game display. The recorded data file stores the display-scaled values, and the scaling factor was divided out before any analysis, so unless otherwise stated all angular values reported in the main manuscript are unscaled physical tilt angles.

Complete Rule Set of the Adaptive Challenge-Penalty Mechanism

The system maintains separate maximum peak thresholds for the forward and backward directions, and challenge levels are tracked independently rather than merged. For a given direction, the mechanism is armed only when the displayed bucket tilt reaches 11° (corresponding to a physical tilt of approximately 18.33° at the foot device); only excursions reaching this arming threshold enter peak updating and penalty-related judgment. Once the movement returns close to the neutral (midline) position, the mechanism disarms for that direction and must reach the 11° threshold again to re-arm. This 11° displayed-angle threshold was determined empirically during system development through iterative testing of the water-flow physical simulation: it is the lower bound at which the displayed bucket can both engage water collection and retain the collected water without spillage. At displayed angles below this threshold the simulation does not initiate collection, whereas at displayed angles substantially above it the inclined bucket loses water before it can fill. The 11° value therefore reflects a playability constraint of the visual game mechanics rather than a biomechanical parameter or an offline signal-analysis criterion.

When a participant reaches the current maximum peak in a given direction, no penalty is triggered if the movement can be maintained at ≥95% of that peak. If this sustained criterion is not met, the penalty mechanism is triggered and the bucket returns toward the midline in small, frequent increments of 0.1° every 0.1 second, producing a smooth, gradual return rather than an abrupt movement. If the participant reaches the current maximum peak on two consecutive occasions and maintains the movement within 95%–100% of that peak both times without triggering the penalty, the system increases the maximum peak threshold for that direction by 0.3° (displayed angle). This increment was designed to encourage mild progression and probe movement boundaries without making the task abruptly too difficult.

After the maximum peak threshold is increased by 0.3°, the system enters a 7-second observation window to continue evaluating the movement state of the participant. If the participant maintains the new peak at ≥95% throughout this window without triggering the penalty mechanism, the new threshold is formally accepted. If the penalty mechanism is triggered during the window, that is, if the participant fails to maintain the required amplitude and the bucket begins to return, and if this penalty-triggered state lasts for more than 4 seconds, the temporary 0.3° increase is canceled and the maximum peak threshold is restored to the previous value.

The penalty-related return of the bucket was applied only to the on-screen visual angle of the bucket for feedback purposes; the tilt-angle signal recorded for analysis always corresponded to the actual (uncorrected) device tilt of the participant and did not include the penalty offset. The mechanism thus applies a penalty only through the visual return of the bucket toward the midline; it does not change the speed of the water flow and does not impose any additional score penalty beyond the basic game feedback.

Table S1. Parameter values of the adaptive challenge-penalty mechanism.

**Parameter**	**Value**	**Scale and role**
Arming threshold (minimum valid peak)	11°	Displayed angle; approximately 18.33° of physical device tilt. Determined empirically from the water-flow simulation; a playability constraint, not a biomechanical or analysis criterion.
Maintenance criterion	≥95% of current peak	Proportion of the current directional maximum peak that must be sustained to avoid a penalty.
Threshold increment	0.3°	Displayed angle; applied after two consecutive qualifying excursions.
Penalty return rate	0.1° every 0.1 s	Displayed angle; produces a smooth, gradual return of the bucket toward the midline.
Observation window	7 s	Period after an increment during which the new peak must be maintained at ≥95% for the increment to be accepted.
Penalty cancellation window	4 s	If the penalty-triggered state persists longer than this, the 0.3° increment is canceled and the previous threshold restored.

Note: All angular values are displayed (on-screen) angles rather than physical device-tilt angles.

Stage 1 Inferential Results

The main manuscript reports the principal Stage 1 inferential findings for both groups. Tables S2 to S5 give the complete repeated-measures ANOVA output, including the Mauchly test result for every outcome and the Greenhouse-Geisser correction where it was applied, together with the Bonferroni-corrected post hoc comparisons and the Friedman sensitivity analyses. All amplitude values are on the physical angle scale.

Table S2. Repeated-measures ANOVA results for the older-adult group (n=20).

Outcome	W	Mauchly P	εGG	F	df1	df2	P	ηp²
Typical forward amplitude	0.9023	0.3966	—	4.169	2.000	38.000	0.023	0.180
Typical backward amplitude	0.5432	0.0041	0.686	4.338	1.373	26.084	0.036	0.186
Forward holding proportion	0.9627	0.7102	—	3.122	2.000	38.000	0.056	0.141
Backward holding proportion	0.9832	0.8587	—	2.563	2.000	38.000	0.090	0.119
Overspeed proportion	0.8694	0.2837	—	0.416	2.000	38.000	0.663	0.021

Note: Greenhouse-Geisser corrected values are shown when the sphericity assumption was violated; a dash indicates that correction was not required. W: Mauchly test statistic; εGG: Greenhouse-Geisser epsilon; df1 and df2: numerator and denominator degrees of freedom; ηp²: partial eta squared.

Table S3. Bonferroni-corrected post hoc paired comparisons for the outcomes of the older-adult group demonstrating nominally significant trial effects (uncorrected P<.05).

Outcome	Comparison	Mean difference (95% CI)	t	df	P	Padj	dz
Typical forward amplitude	Trial 1 vs Trial 2	1.714 (0.11 to 3.32)	2.241	19	0.0372	0.1116	0.501
	Trial 2 vs Trial 3	0.108 (−1.13 to 1.34)	0.183	19	0.8567	>.99	0.041
	Trial 1 vs Trial 3	1.822 (0.24 to 3.41)	2.405	19	0.0265	0.0795	0.538
Typical backward amplitude	Trial 1 vs Trial 2	1.824 (0.74 to 2.91)	3.518	19	0.0023	0.0069	0.787
	Trial 2 vs Trial 3	0.748 (−1.35 to 2.84)	0.748	19	0.4635	>.99	0.167
	Trial 1 vs Trial 3	2.572 (0.32 to 4.82)	2.395	19	0.0271	0.0813	0.535

Note: Mean differences are reported as later trial minus earlier trial. Adjusted P values that reached the upper bound of the Bonferroni correction are reported as >.99. CI: confidence interval; df: degrees of freedom; Padj: Bonferroni-adjusted P value; dz: within-participant standardized mean difference.

Table S4. Friedman sensitivity analyses for outcomes exhibiting deviations from normality.

Group	Outcome	χ²	df	P	Kendall's W
Older-adult group	Typical backward amplitude	9.30	2	0.0096	0.233
Younger-adult group	Typical forward amplitude	1.90	2	0.3867	0.048
Younger-adult group	Overspeed proportion	1.30	2	0.5220	0.033

Note: χ²: Friedman test statistic; df: degrees of freedom; Kendall's W: Kendall coefficient of concordance.

Table S5. Repeated-measures ANOVA results for the younger-adult group (n=20).

Outcome	W	Mauchly P	εGG	F	df1	df2	P	ηp²
Typical forward amplitude	0.3786	0.0002	0.617	0.803	1.234	23.437	0.404	0.041
Typical backward amplitude	0.9302	0.5214	—	1.232	2.000	38.000	0.303	0.061
Forward holding proportion	0.9122	0.4375	—	1.725	2.000	38.000	0.192	0.083
Backward holding proportion	0.9021	0.3956	—	0.234	2.000	38.000	0.793	0.012
Overspeed proportion	0.7878	0.1169	—	0.326	2.000	38.000	0.724	0.017

Note: Greenhouse-Geisser corrected values are shown when the sphericity assumption was violated; a dash indicates that correction was not required. W: Mauchly test statistic; εGG: Greenhouse-Geisser epsilon; df1 and df2: numerator and denominator degrees of freedom; ηp^2: partial eta squared.

Stage 2 Inferential Results

Table S6 gives the complete repeated-measures ANOVA results for the seven signal-derived measures examined by difficulty condition and presentation position in the Stage 2 counterbalanced supplementary study.

Table S6. Repeated-measures ANOVA results for difficulty condition and presentation position in the Stage 2 counterbalanced supplementary study (n=18).

Outcome	Difficulty condition F(2,34)	P	ηp²	Presentation position F(2,34)	P	ηp²
Typical forward amplitude	0.746	0.482	0.042	0.315	0.732	0.018
Typical backward amplitude	1.413	0.257	0.077	1.261	0.296	0.069
Forward holding proportion	1.307	0.284	0.071	0.357	0.702	0.021
Backward holding proportion	1.674	0.203	0.090	0.582	0.564	0.033
Forward effective flexion-extension proportion	0.759	0.476	0.043	2.162	0.131	0.113
Backward effective flexion-extension proportion	0.409	0.667	0.024	1.927	0.161	0.102
Overspeed proportion	1.083	0.350	0.060	0.169	0.845	0.010

Note: Repeated-measures ANOVAs were conducted separately for difficulty condition and presentation position. The numerator and denominator degrees of freedom were 2 and 34, respectively, for all tests. ηp^2: partial eta squared.

This is a Multimedia Appendix to a manuscript published in JMIR Serious Games. For full copyright and citation information, see the published article
