# Supplementary material for: Balance Wood, a Bilateral Ankle-Controlled Serious Game for Healthy Adults: 2-Stage Cross-Sectional Development and Usability Study
Source: JMIR Serious Games. 2026 Sep 25;14:e96617. doi: 10.2196/96617 (PMC13614508; doi:10.2196/96617)
Supplement: Supplementary file 2 [file games-v14-e96617-s004.pdf]

# Balance Wood System Evaluation Questionnaire

Participant ID: ____________ Date: ____________ Trial order: ____________

For each item, please circle one response option.

## Part 1. Overall usability of the system

Please answer intuitively on a scale from 1 (Strongly disagree) to 5 (Strongly agree). Note: the statements alternate between positive and negative wording, so please read each one carefully before answering.

| Item | Strongly disagree | Disagree | Neither | Agree | Strongly agree |
|---|---|---|---|---|---|
| 1. I think that I would like to use this system frequently. | ○ | ○ | ○ | ○ | ○ |
| 2. I found this system unnecessarily complex. | ○ | ○ | ○ | ○ | ○ |
| 3. I thought this system was easy to use. | ○ | ○ | ○ | ○ | ○ |
| 4. I think I would need the help of a knowledgeable person to use this system. | ○ | ○ | ○ | ○ | ○ |
| 5. I felt the various functions of this system were well integrated. | ○ | ○ | ○ | ○ | ○ |
| 6. I felt there was a lack of consistency in how this system is operated. | ○ | ○ | ○ | ○ | ○ |
| 7. I think most people would learn to use this system very quickly. | ○ | ○ | ○ | ○ | ○ |
| 8. I found this system cumbersome to operate. | ○ | ○ | ○ | ○ | ○ |
| 9. I felt confident when operating this system. | ○ | ○ | ○ | ○ | ○ |
| 10. I needed to learn a lot of things before I could get going with this system. | ○ | ○ | ○ | ○ | ○ |

## Part 2. Your impressions of actually using Balance Wood

Please circle one response option from 1 (Strongly disagree) to 5 (Strongly agree).

| **Item** | Strongly disagree | Disagree | Neither | Agree | Strongly agree |
|---|---|---|---|---|---|
| 1. From the visual feedback (water spilling, the direction the bucket tilts), I could intuitively understand the purpose of the task. | ○ | ○ | ○ | ○ | ○ |
| 2. The response that water spills when switching too quickly between ankle dorsiflexion and plantarflexion helped me adjust the speed of my movement. | ○ | ○ | ○ | ○ | ○ |
| 3. Over the course of the three trials, I felt fatigue in my ankle. | ○ | ○ | ○ | ○ | ○ |
| 4. I felt concerned about safety during operation (e.g., my posture became unstable, or the chair felt like it might tip over). | ○ | ○ | ○ | ○ | ○ |
| 5. I experienced pain or discomfort during operation. | ○ | ○ | ○ | ○ | ○ |
| 6. I found this game enjoyable. | ○ | ○ | ○ | ○ | ○ |
| 7. I would have no objection to using this system regularly (e.g., 2–3 times per week). | ○ | ○ | ○ | ○ | ○ |
| 8. When tilting the bucket, I felt I was mainly using ankle movement. | ○ | ○ | ○ | ○ | ○ |
| 9. When tilting the bucket, I felt I was also using parts of the body other than the ankle. | ○ | ○ | ○ | ○ | ○ |

## Part 3. Body parts you consciously used during operation (3 classifications)

Regarding the body parts you consciously used during operation, please rate the degree of use from each of the following three perspectives (A: joints, B: muscles, C: body regions), from 1 (Not at all) to 5 (Very much).

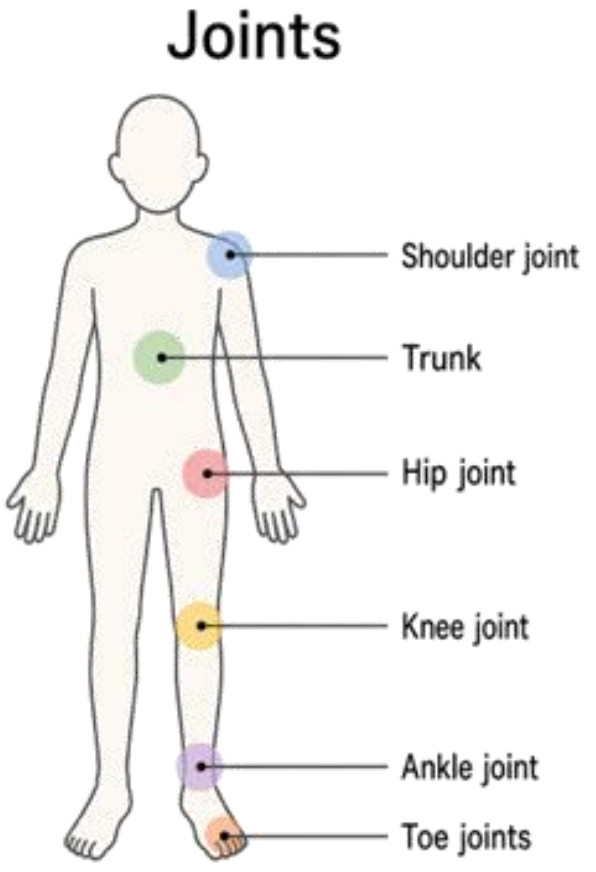


| Part | Not at all | A little bit | Somewhat | Quite a bit | Very much |
|---|---|---|---|---|---|
| Ankle joint | ◯ | ◯ | ◯ | ◯ | ◯ |
| Knee joint | ◯ | ◯ | ◯ | ◯ | ◯ |
| Hip joint | ◯ | ◯ | ◯ | ◯ | ◯ |
| Trunk | ◯ | ◯ | ◯ | ◯ | ◯ |
| Shoulder joint | ◯ | ◯ | ◯ | ◯ | ◯ |
| Toe joints | ◯ | ◯ | ◯ | ◯ | ◯ |

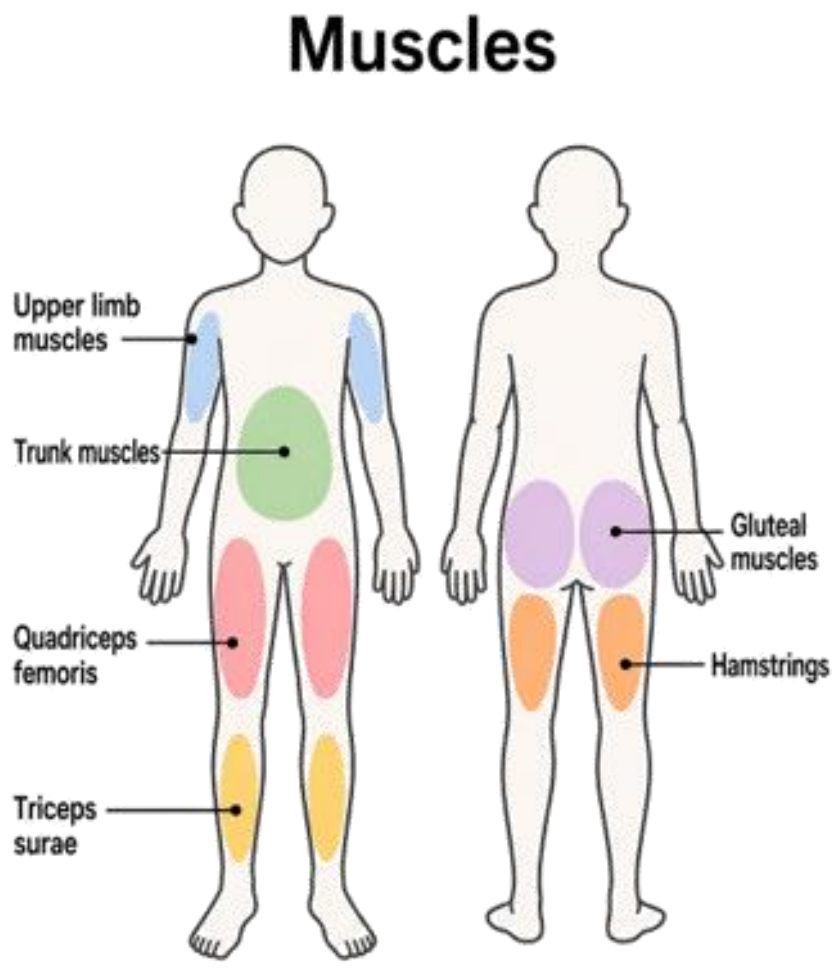


| Part | Not at all | A little bit | Somewhat | Quite a bit | Very much |
|---|---|---|---|---|---|
| Triceps surae | ◯ | ◯ | ◯ | ◯ | ◯ |
| Quadriceps femoris | ◯ | ◯ | ◯ | ◯ | ◯ |
| Hamstrings | ◯ | ◯ | ◯ | ◯ | ◯ |
| Gluteal muscles | ◯ | ◯ | ◯ | ◯ | ◯ |
| Trunk muscles | ◯ | ◯ | ◯ | ◯ | ◯ |
| Upper limb muscles | ◯ | ◯ | ◯ | ◯ | ◯ |

## Body Regions

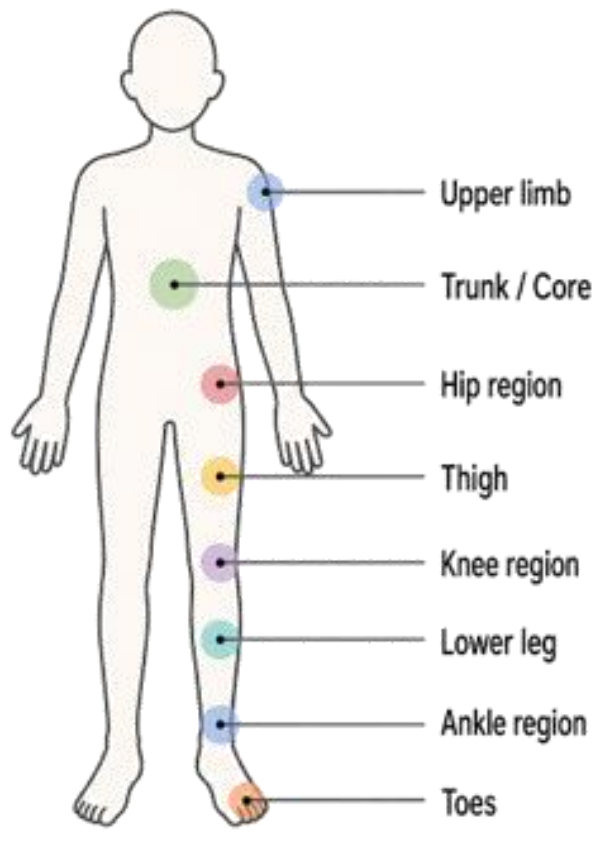


| Part | Not at all | A little bit | Somewhat | Quite a bit | Very much |
|---|---|---|---|---|---|
| Ankle region | ◯ | ◯ | ◯ | ◯ | ◯ |
| Lower leg | ◯ | ◯ | ◯ | ◯ | ◯ |
| Knee region | ◯ | ◯ | ◯ | ◯ | ◯ |
| Thigh | ◯ | ◯ | ◯ | ◯ | ◯ |
| Hip region | ◯ | ◯ | ◯ | ◯ | ◯ |
| Trunk / Core | ◯ | ◯ | ◯ | ◯ | ◯ |
| Upper limb | ◯ | ◯ | ◯ | ◯ | ◯ |
| Toes | ◯ | ◯ | ◯ | ◯ | ◯ |

## Open comments

Please feel free to write any impressions, suggestions for improvement, or anything else you noticed about this system or task.

**Thank you very much for your cooperation.**

# Balance Wood システム評価 質問紙

参加者 ID: ______________ 日付: ______________ 試行順序: ______________

各項目について、最もあてはまる番号に○をつけてください。

## システム全体の使いやすさについてお伺いします

1（まったくそう思わない）から 5（強くそう思う）の中から、直感的にお答えください。※ 質問には肯定的な表現と否定的な表現が交互に含まれています。一問ずつよく読んでからお答えください。

| 項目 | まったくそう思わない | あまりそう思わない | どちらでもない | ややそう思う | 強くそう思う |
|---|---|---|---|---|---|
| 1. このシステムを日常的に使いたいと思った | ○ | ○ | ○ | ○ | ○ |
| 2. このシステムには不必要に複雑な部分があると感じた | ○ | ○ | ○ | ○ | ○ |
| 3. このシステムは簡単に操作できると思った | ○ | ○ | ○ | ○ | ○ |
| 4. このシステムを使うには詳しい人の助けが必要だと思った | ○ | ○ | ○ | ○ | ○ |
| 5. このシステムの各機能はうまくまとまっていると感じた | ○ | ○ | ○ | ○ | ○ |
| 6. このシステムの操作方法に統一感がないと感じた | ○ | ○ | ○ | ○ | ○ |
| 7. ほとんどの人がこのシステムをすぐに使えるようになると思った | ○ | ○ | ○ | ○ | ○ |
| 8. このシステムは操作が面倒だと感じた | ○ | ○ | ○ | ○ | ○ |
| 9. このシステムを使うとき、自信を持って操作できた | ○ | ○ | ○ | ○ | ○ |
| 10. このシステムを使い始める前に、覚えることが多かった | ○ | ○ | ○ | ○ | ○ |

## Balance Wood を実際に使ってみたご感想についてお伺いします

1（まったくそう思わない）から 5（強くそう思う）の中から○をつけてください。

| 項目 | まったく<br>そう思わない | あまり<br>そう思わない | どちらでもない | やや<br>そう思う | 強く<br>そう思う |
|---|---|---|---|---|---|
| 1. 視覚的フィードバック（水のこぼれ、バケツの傾く方向）から、タスクの目的を直感的に理解できた | ○ | ○ | ○ | ○ | ○ |
| 2. 足首の背屈・底屈の切り替えが速すぎると水がこぼれるという反応は、動きの速さを調整するのに役立った | ○ | ○ | ○ | ○ | ○ |
| 3. 3 つの試行を通じて、足首に疲れを感じた | ○ | ○ | ○ | ○ | ○ |
| 4. 操作中に体勢が不安定になる、椅子が傾きそうになるなど、安全面で不安を感じた | ○ | ○ | ○ | ○ | ○ |
| 5. 操作中に痛みや不快感があった | ○ | ○ | ○ | ○ | ○ |
| 6. このゲームは楽しいと感じた | ○ | ○ | ○ | ○ | ○ |
| 7. このシステムを定期的（例：週 2〜3 回）に使うことに抵抗はない | ○ | ○ | ○ | ○ | ○ |
| 8. バケツを傾けるとき、主に足首の動きを使っていると感じた | ○ | ○ | ○ | ○ | ○ |
| 9. バケツを傾けるとき、足首以外の部位も使っていると感じた | ○ | ○ | ○ | ○ | ○ |

## 操作中に意識的に使った身体部位についてお伺いします（3 分類）

操作中に意識的に使った身体部位について、以下の 3 つの観点（A: 関節、B: 筋肉、C: 身体部位）でそれぞれの使用程度を、1（まったく使わなかった）から 5（強く使った）の中から〇をつけてください。

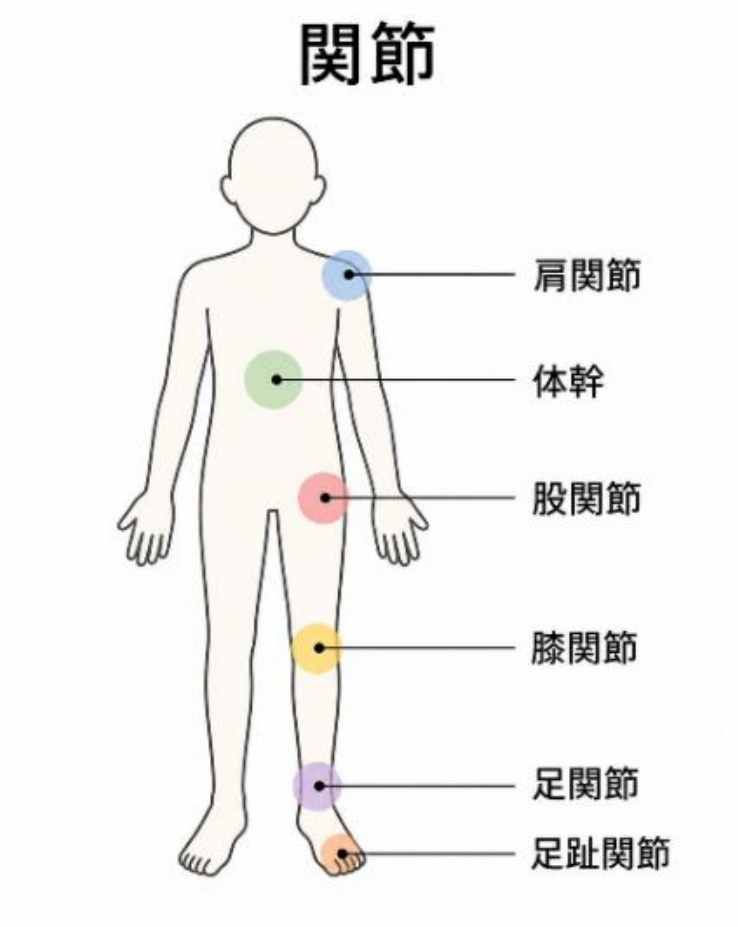


| 部位 | まったく<br>使わなかった | あまり<br>使わなかった | どちらでもない | やや使った | 強く使った |
|---|---|---|---|---|---|
| 足関節 | 〇 | 〇 | 〇 | 〇 | 〇 |
| 膝関節 | 〇 | 〇 | 〇 | 〇 | 〇 |
| 股関節 | 〇 | 〇 | 〇 | 〇 | 〇 |
| 体幹 | 〇 | 〇 | 〇 | 〇 | 〇 |
| 肩関節 | 〇 | 〇 | 〇 | 〇 | 〇 |
| 足趾関節 | 〇 | 〇 | 〇 | 〇 | 〇 |

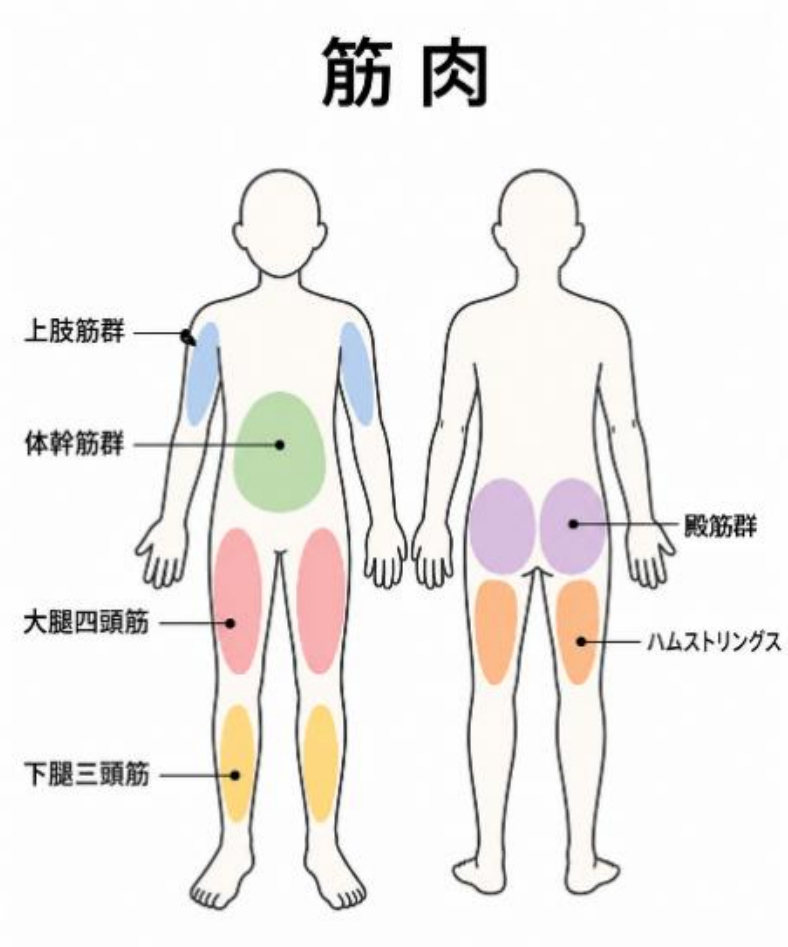


| 部位 | まったく<br>使わなかった | あまり<br>使わなかった | どちらでもない | やや使った | 強く使った |
|---|---|---|---|---|---|
| 下腿三頭筋 | 〇 | 〇 | 〇 | 〇 | 〇 |
| 大腿四頭筋 | 〇 | 〇 | 〇 | 〇 | 〇 |
| ハムストリングス | 〇 | 〇 | 〇 | 〇 | 〇 |
| 殿筋群 | 〇 | 〇 | 〇 | 〇 | 〇 |
| 体幹筋群 | 〇 | 〇 | 〇 | 〇 | 〇 |
| 上肢筋群 | 〇 | 〇 | 〇 | 〇 | 〇 |

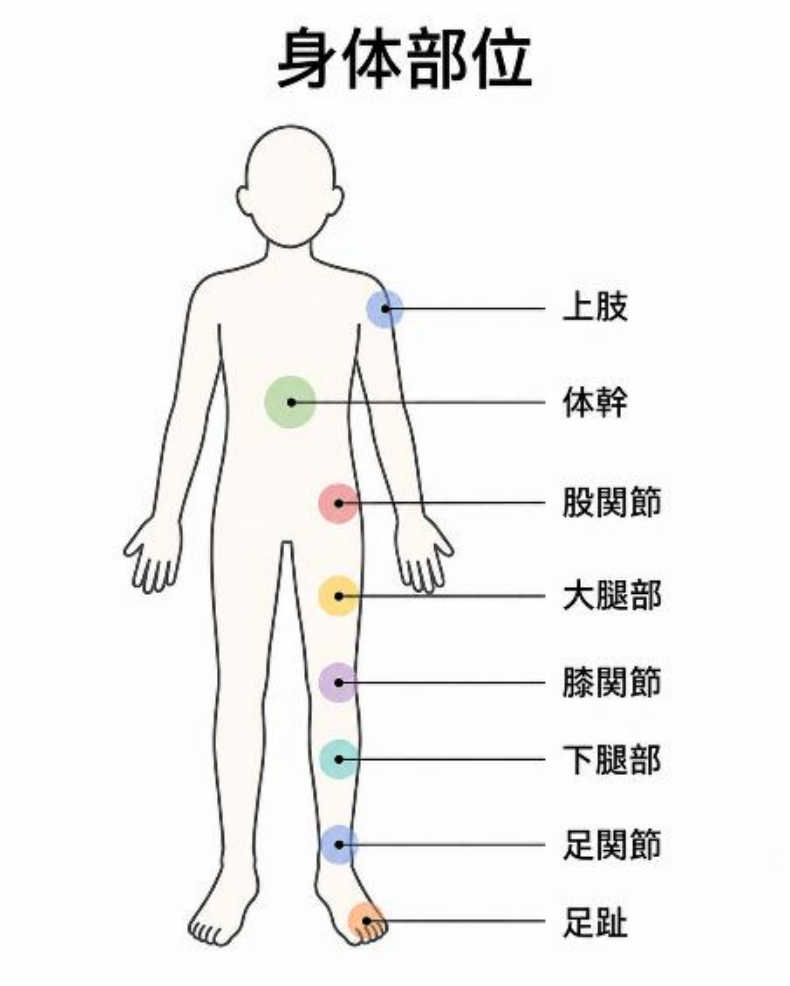


| 部位 | まったく使わなかった | あまり使わなかった | どちらでもない | やや使った | 強く使った |
|---|---|---|---|---|---|
| 足関節 | ◯ | ◯ | ◯ | ◯ | ◯ |
| 下腿部 | ◯ | ◯ | ◯ | ◯ | ◯ |
| 膝関節 | ◯ | ◯ | ◯ | ◯ | ◯ |
| 大腿部 | ◯ | ◯ | ◯ | ◯ | ◯ |
| 股関節 | ◯ | ◯ | ◯ | ◯ | ◯ |
| 体幹 | ◯ | ◯ | ◯ | ◯ | ◯ |
| 上肢 | ◯ | ◯ | ◯ | ◯ | ◯ |
| 足趾 | ◯ | ◯ | ◯ | ◯ | ◯ |

## ご自由にお書きいただける欄を設けました

本システムや本タスクについて、感想・改善提案・気づいた点などがあれば、ご自由にお書きください。

ご協力ありがとうございました。
